# Analysis of the Effect of the Biomass Torrefaction Process on Selected Parameters of Dust Explosivity

**DOI:** 10.3390/molecules25153525

**Published:** 2020-08-01

**Authors:** Marcin Bajcar, Bogdan Saletnik, Grzegorz Zaguła, Czesław Puchalski

**Affiliations:** Department of Bioenergetics, Food Analysis and Microbiology, Institute of Food Technology and Nutrition, College of Natural Science, Rzeszow University, Ćwiklińskiej 2D, 35-601 Rzeszow, Poland; mbajcar@ur.edu.pl (M.B.); g_zagula@ur.edu.pl (G.Z.); cpuchal@ur.edu.pl (C.P.)

**Keywords:** lignocellulosic biomass, torrefaction, explosivity, dust

## Abstract

This article presents the findings of a study investigating the explosion and combustion parameters of dust from the raw biomass of wheat straw and energy willow and from the products of biomass torrefaction generated at temperatures ranging from 220 to 300 °C. Agricultural waste and energy crops and their modifications, e.g., in the torrefaction process, did not find a place in explosive risk research, which the authors decided to present in their work. The study was designed to estimate explosion hazard during the processing of the materials into fuels and during the storage process. The measurements recorded a maximum explosion pressure P_max_ in the case of dust from biomass ranging from 7.2 to 7.3 bar and for dust from torrefied materials amounting to 7.5–9.2 bar, and a maximum rate of pressure rise over time (*dp/dt*)_max_ in raw biomass ranging from 201.4 to 261.3 bar/s and in torrefied materials amounting to 209.6–296.6 bar/s. The estimated explosion index *K_st_*_max_ for raw biomass was 55–72 m*bar/s and for torrefied materials was in the range from 57 to 81 m*bar/s. In the results, the authors present values for specific types of fuel which differ significantly depending on the type of biomass. The research findings show that the torrefaction process used in fuel production is not associated with a significantly greater risk of explosion and the materials obtained may safely be used as an alternative to conventional solid fuels. Given the growing interest in the use of biomass and in the variety of biomass processing methods for energy-related purposes, it seems there is a need for research to develop appropriate guidelines and for effective practices to be introduced in the energy industry in order to ensure the safety of the processes used in the production of novel fuels especially in small installations converting these materials into more efficient energy material.

## 1. Introduction

In recent years, energy security has become a key factor in the economic growth of many countries [1,2]. Energy companies and organizations specializing in energy production are continuously looking for new solutions which will permit the development of ecologically sustainable, effective, and safe methods of obtaining energy [3]. It is obviously necessary to ensure a continuity of energy supply. It will be possible to achieve this goal if energy systems are constantly modernized and if a range of different energy sources are used. In structural terms, however, this goal is realized by power industries worldwide in different ways. There are many countries which use a single energy source, predominantly coal, with only a marginal contribution from additional resources [4]. It is necessary to seek out resources to enable the development of more environmentally friendly mixed models. Undoubtedly, the use of renewable energy sources such as plant biomass constitutes an alternative to conventional energy systems [5]. In energy systems which take advantage of the potential lying in biomass-coal co-combustion or in technologies for the physical or thermal processing of biomass, it is necessary to develop detailed rules and guidelines related to the safe operation and use of the energy infrastructure applying these technologies [6].

Torrefaction is one of the methods that enhance biomass for purposes related to energy production. This thermal processing technology is mainly applied to biomass originating from plants, i.e., to lignocellulosic biomass, to transform it into a form of solid fuel. Products of torrefaction have properties similar to those of bituminous coal [7,8]. The temperatures applied in the process are in the range from 200 to 300 °C and torrefaction is carried out in a neutral anaerobic atmosphere under a pressure similar or equal to atmospheric pressure [9,10]. Changes in the biomass structure result from the destruction of its fibrous backbone induced by the decomposition of polymer structures; this leads to a decrease in the mechanical strength of the material obtained which as a result makes it possible to reduce the costs connected with preparation of the final form of the fuel, e.g., pellets [11]. In a typical torrefaction process the material is partly degassed, which leads to weight loss, however the simultaneous decrease in energy content is lower than the weight loss [12]. This biomass processing technology leads to a 30% decrease in weight, while preserving 90% of the original energy content [13].

Plant biomass is a specific type of material which in the course of the processing is usually refined into smaller fractions, as a result producing a significant amount of dust, adversely affecting the production area and equipment [14,15]. This is a dangerous phenomenon which in some circumstances may lead to an explosion or the self-ignition of dust, and therefore it is associated with a high fire risk. Explosion hazards are known in many energy technologies [16]. A variety of procedures to provide protection against explosion have been developed over the years. However, protection plans have commonly been developed for large installations, while in recent years a noticeable increase in the number of micro-installations has been seen in addition to the new fuels being introduced in the prosumer energy sector. Given this, there is a continued need for related research [17].

We should remember that there are many risk factors necessary for self-ignition of dust to occur. Specific conditions must be met for the phenomenon to take effect [18]. The factors necessary for the explosion of a dust cloud to take place, referred to as the “explosion pentagon”, include fuel in the form of dust particles, oxidant, an ignition source, mixing, as well as confinement [19]. One of the related risks is associated with the direct contact of dust with hot surfaces. In technological lines this occurs in locations connected with the transformation of electrical and mechanical energy into thermal energy, e.g., electric motors, transmission belts, bearings, etc. Incorrect use of these elements results in a significant increase in their temperature [20]. Other risk factors, often directly linked to biomass processing technologies, include hot gases produced during the process, which pose a hazard if they come into direct contact with a dust–air mixture [21]. Users of installations should pay particular attention to adequate protection against the access of foreign bodies into devices carrying the IP sign. If insufficiently protected, such devices may generate mechanical and electrical sparks which may initiate an explosion [22]. Frequent problems encountered during the processing of lignocellulosic materials include electrostatic discharges. These are most commonly induced by materials coming in contact as a result of friction during such processes as milling, as well as the crushing of materials. Such phenomena commonly occur during the processing and storage of biomass [23,24,25]. This is also facilitated by biomass transport systems, such as pneumatic pipes, belt conveyors, dispensers, and fractioning systems. The operator may also be exposed to electrostatic discharge, due to which it is necessary for them to use adequate protection [26,27]. 

Given the growing interest in the use of biomass and in the variety of methods of processing biomass for energy-related purposes, it seems there is a need for research to develop appropriate guidelines to be put into effect in the energy industry to ensure the safety of the processes used in the production of novel fuels. The purpose of the present study was to identify the risk of explosion during the processing of plant biomass into solid fuels and during its storage.

## 2. Results and Discussion

The development of industry significantly increased the importance of preventing explosions designed to reduce the likelihood or impact of industrial processes. The development of technical solutions is necessarily associated with conducting these studies focused on broader knowledge of the basic course of explosion characteristics and mechanisms of action of its determinants. The results of this type of research are thoroughly analyzed due to the number of factors determining the course of dust explosion. This study investigated biomass from energy willow and wheat straw. Willow is one of the most frequently cultivated energy crops, while wheat straw, classified as agricultural waste biomass, is obtained from plant production practically all over the world and is therefore widely available, as has been shown by Węgrzyn and Zając in their study [28]. In order to classify the materials, raw biomass and torrefaction products were subjected to thermogravimetric and calorimetric assessment and were examined for elemental composition. The results are presented in Table 1. Analysis of the specific parameters shows that the torrefaction process leads to increased contents of carbon and volatile substances and to a greater calorific value of the material. Similar findings were reported by Chin et al., Poundel et al., and Werkelin et al. [29,30,31]. A comparative analysis of the raw biomass and torrefied materials shows significant differences resulting from the temperature applied during the process. These in turn significantly affect the explosion parameters of these materials, as shown by the results of these analyses. 

The presentation of the study results applies specific abbreviations to the specific research materials (see Abbreviations). 

The results of laboratory tests, presented as mean values from three measurements, are shown in Table 2. The analyses took into account the maximum explosion pressure P_max_ in biomass which was not thermally processed and in torrefied materials. The findings showed that the value of P_max_ ranged from 7.3 bar in the case of raw willow to 9.2 bar in torrefied material generated from willow at 300 °C. A similar tendency, yet with visibly lower dynamics of increase, was observed in the case of wheat straw; more specifically the maximum explosion pressure identified in raw wheat straw was at the level of 7.3 bar, and the highest value of this parameter, 8.8 bar, was measured in the torrefied material produced at 300 °C. The observed tendency for an increase is associated with changes in the composition and physical structure of the material. The torrefaction process leads to an increased concentration of carbon, higher contents of volatile substances, and greater brittleness observed in the torrefied materials. The difference in the quantity of volatile substances according to the authors is due to the method of preparation of test material. After harvesting, the material was left to air-dry, and then processed in further analyses. Thus, a more increase of the amount of volatile substances relative to the raw biomass was observed in torrefied material.

A similar tendency was observed in measurements assessing the maximum rate of pressure rise. The lowest values were identified in the raw biomass, and an increase in this parameter that was measured in the torrefied materials corresponded to the higher temperatures of the process. The torrefied material differs from the raw biomass in terms of the physicochemical characteristics, and this has impacts on the values of this parameter. Despite the visible trend, these differences are not significant and do not result in a change of dust classification. According to Cashdollar, Cordero et al., and Demirbas [33,34,35], as well as other researchers, the differences between raw and torrefied biomass can mainly be explained by the different emissivity of the respective materials linked to the mechanisms of heat transfer. A linear relationship was observed between the values of P_max_ and (*dp/dt*)_max_, which was also reported by Torrent et al. and Holbrow [36,37].

Assessment of the lower explosion limit (LEL) showed that the proportion of dust in the raw biomass and in the torrefaction products generated from energy willow at 220–260 °C and from wheat straw at 220–280 °C corresponded to greater LEL values when compared to the materials torrefied at higher temperatures. The difference was twofold in both the willow and wheat straw materials. According to Kok et al., Medina et al., and Regland et al., the variation in levels of this parameter results from a number of physicochemical properties of raw biomass and torrefied materials, such as the size and shape of particles, bulk density, etc., which has also been reported in other studies [38,39,40].

**Figure 1:**
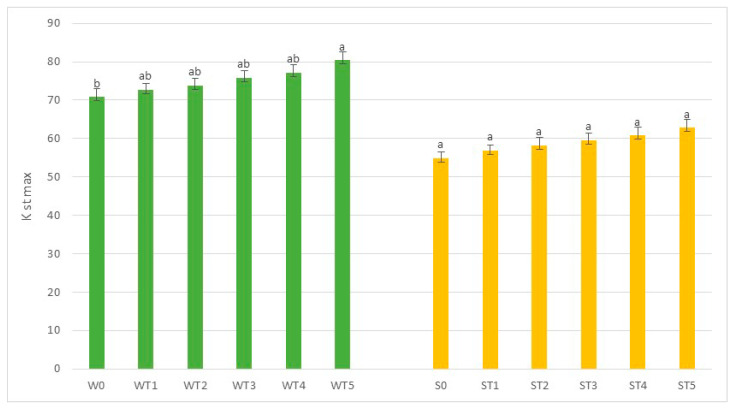
Changes in the dust explosion index in the samples of biomass and torrefied materials. Statistically significant differences between marked by different letters (*p* ≤ 0.05).

The parameter which provides a direct classification of the dust explosion hazard is the explosion index *Kst* max, whose value was calculated in accordance with the relevant standard (PN-EN 14034-1, 2011). Based on the values of the index recorded and shown in Figure 1, it was possible to determine that both the raw biomass and the torrefied materials represent a Class 1 risk of dust explosion, i.e., the class with the lowest dust explosion index. This parameter is a practical answer to the classification of a given material, providing a reference for production guidelines, for adaptation and protection against explosion hazard, and for designing solutions as well as protection and safety systems, which have been discussed in studies by Eckhoff as well as Taveau et al. [41,42].

**Figure 2:**
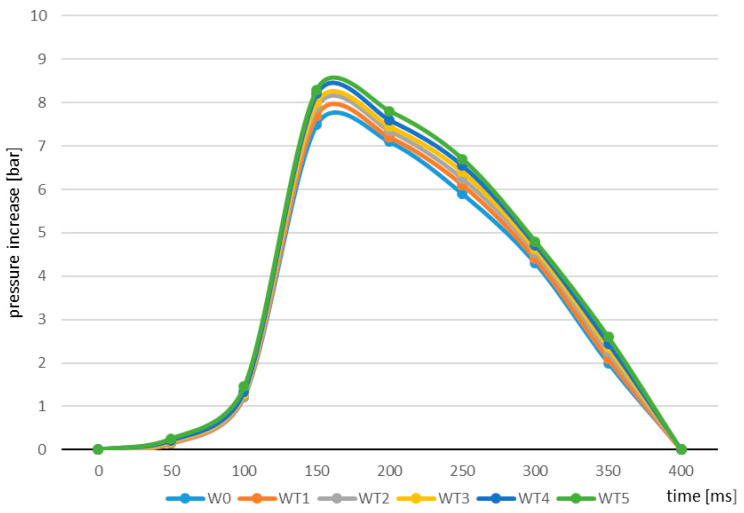
Explosion pressure curve identified for samples of raw and torrefied willow material.

In the case of the dust explosion analyses, the specific indicators are determined taking into account a number of factors impacting the course and dynamics of the processes taking place, e.g., particle structure, content of volatile elements, water in the material, etc. The tests carried out for the needs of the present study made it possible to determine the changes in pressure during the explosion of dust from the raw biomass and the torrefied materials. Figure 2 presents differences in the curves reflecting this parameter relative to the material. Cashdollar and Arnaldos presented similar dependencies and graphs of pressure increase curves for similar groups of materials [33,43]. Pressure intensity and increase occur rapidly, reaching maximum values, followed by a gradual decrease in pressure to the initial value. The course and the dynamics of the process had similar characteristics in both raw and torrefied willow. It was found that an increase in the temperature of the torrefaction process is associated with a greater increase in the maximum pressure compared to raw willow biomass. The highest value of this parameter, exceeding 8.5 bar, was observed in the case of willow biomass torrefied at 300 °C.

**Figure 3:**
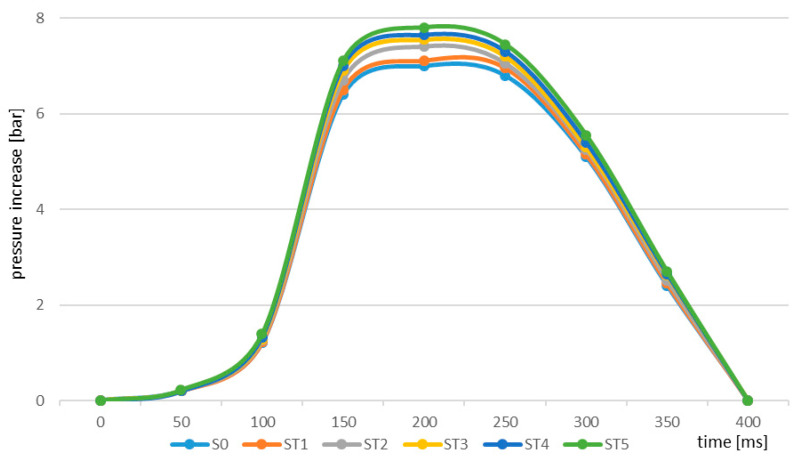
Explosion pressure curve identified for samples of raw and torrefied wheat straw.

Similar dynamics of the changes were identified in the case of wheat straw; however, the rate of maximum increase in pressure was visibly lower in both the raw and the torrefied straw material (Figure 3). The different dynamics of the increase in pressure are associated with variation in the heat of combustion characteristics of the materials. Willow material is more carbonized and produces a greater amount of heat, which was confirmed by thermogravimetric analysis. Materials with a higher carbon content, especially in the volatile phase, cause faster combustion of the material, which is why the explosion dynamics are faster in their case. Of course, this is one of the many elements affecting this effect.

It was found that the impact of initial pressure on the explosive concentration limits is small. The increase in pressure usually increases the range of concentration explosion limits, and while the changes of the lower explosion limit are small, the upper explosion limit changes more. Porowski et al. showed similar relationships in their research [44]. There is often a need to determine the concentration explosion limits of mixtures. Knowing the concentration limits of explosion of the components of the mixture, one can estimate its concentration limits.

## 3. Materials and Methods

### 3.1. Torrefaction Process

Comprehensive laboratory tests assessing the impact of the torrefaction process on changes in the explosive properties of dust in the process of generating torrefaction products took into account straw from a winter variety of wheat and energy willow harvested in a three-year cycle. After it was collected, the raw material designated for the study was brought to an air-dry state, and then subjected to grinding. Material with a total weight of 10 kg of willow and 10 kg of straw was prepared for laboratory tests. Then, three replicates were performed for each type of test and 19 samples were placed in each test. The torrefaction process was carried out in a LECO TGA 701 stationary bed reactor (Leco, St. Joseph, MI, USA). Examination of the physicochemical properties and torrefaction tests with the use of a thermobalance were carried out on the feedstock with a grain size below 10 mm. At work, only fragmented material was tested. It took into account all dust fractions of particle sizes occurring in biomass processing, and also met the requirements for material preparation in buckling analyzes. The material was not pelleted. Samples of energy willow and wheat straw were subjected to a torrefaction process at 220, 240, 260, 280, and 300 °C for a duration of 60 min in a nitrogen atmosphere with 99.99% purity, at a gas flow rate of 10 L/min, and with temperature increases of 30 °C/min. 

### 3.2. Samples Analysis

The material was examined for its basic physicochemical parameters, such as total content of carbon, ash, nitrogen, hydrogen, water, and volatile substances as well as its calorific value. The assessments were carried out using a LECO TGA 701 thermogravimetric analyzer, a truespec LECO CHN elemental composition analyzer (Leco, St. Joseph, MI, USA), and a LECO AC 500 isoperibolic calorimeter (Leco, St. Joseph, MI, USA). 

**Figure 4:**
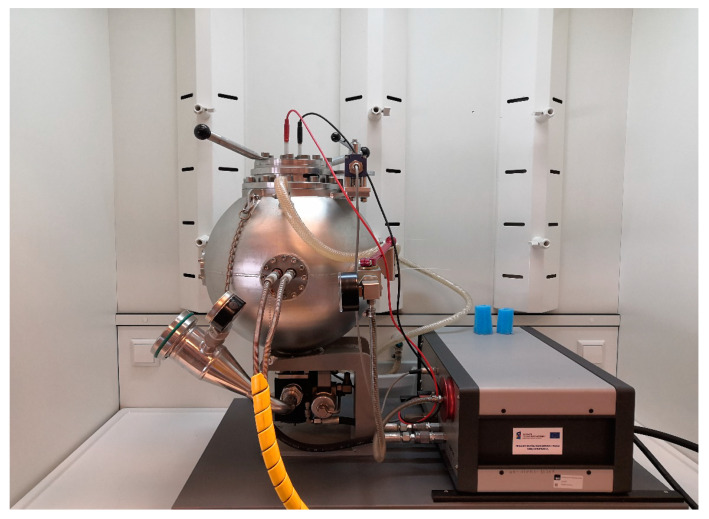
KSEP 310 explosivity analyzer.

After analysis of the qualitative assessment of raw biomass and torrefunctions, 100 g samples were prepared for each type of material. The material was ground in a ball mill to a dust fraction with a particle diameter smaller than 1 mm. The material prepared in this way was intended for explosion tests. Explosion tests were carried out in triplicate. Explosivity tests were performed using a KSEP20 device, with a KSEP 310 control unit (Kuhner AG, Basel, Switzerland). The test chamber is a sphere with a volume of 20 dm^3^. The chamber has a water jacket designed to dissipate the heat of explosions and maintain thermostatically controlled test temperatures (Figure 4).

The dust designated for testing is dispersed under pressure with the use of an outlet valve which opens and closes pneumatically. The ignition source consists of two chemical ignitors, each with an energy rating of 5 kJ, located centrally in the sphere. The changing process parameters are recorded by means of Kistler piezoelectric pressure sensors. The measurements identified the maximum explosion pressure P_max_, defined as the highest registered pressure during explosion of a flammable mixture consisting of a combustible material and air. This parameter is used along with the value of the maximum rate of pressure rise over time (*dp/dt*)_max_, to determine the explosion class *K_st_*_max_. The latter parameter, providing the basis for European standards, is a measure determining the classification of combustible dust according to PN-EN 14034-2 [45]. It is estimated based on the following formula,

(1) $$K\max=Kst=\sqrt[{3}]{V(\frac{dp}{dt}})\max=0.271(\frac{dp}{dt})\max[{\mathrm{mbars}}^{-1}]$$

where

*K_st_*_max_—explosion index,

*V*—volume of test chamber, and

(*dp/dt*)_max_—maximum rate of explosion pressure rise.

Explosion indices were classified in accordance with the values shown in Table 3, where the St 1 Class refers to materials presenting low explosion hazard, the St 2 Class to materials with a moderate risk of explosion, and the St 3 Class to materials presenting a high risk of explosion.

### 3.3. Statistical Analysis

The effects of the experimental factors reflected by the relevant parameters, and the relationships between these, were examined using Analysis of Variance (ANOVA) by means of the Duncan test. Statistica 12 software was applied to compute the statistical analyses. A significance threshold of ≤0.05 was set for all analyses. The data were analyzed separately for energy willow and wheat straw.

## 4. Conclusions

Biomass is a flammable material which has been used in various energy systems worldwide for many years to great effect. Basic research focusing on its physicochemical characteristics comprises a variety of studies investigating various types of biomass and fuels generated through thermochemical modifications. Indeed, the properties of raw biomass have been extensively examined and reported. On the other hand, the products of biomass processing continue to be investigated in detail and there are still insufficient data to describe their properties e.g., related to the safety of the processing operations and the use of these fuels for energy-related purposes. In the work, the authors presented a method of preparing samples typical for small installations, often home solutions, which do not have an extensive technological line, and the material after harvest is usually stored for a long time in order to reduce the water content. In addition, agricultural materials and energy crops, which are the main elements of substrates in small installations, are described less frequently than forest materials. The current findings showed an increase in the explosion index *K_st_*_max_, which, in the case of raw willow biomass, was estimated at the level of 72 m*bar/s, and for the torrefied material amounted to 81 m*bar/s. A similar tendency was identified in the case of wheat straw; the dust explosion index *K_st_*_max_ of raw biomass amounted to 55 m*bar/s, and with the torrefied materials it increased to 62 m*bar/s. The present study shows that modifications of raw biomass required for the production of fuels with better quality parameters do not increase the risk of explosion. Furthermore, the ultimate gain in the quality of the fuel thus obtained justifies further research into this group of fuels derived from plant biomass.

## Figures and Tables

**Table 1:** The parameters identified for raw biomass and material torrefied for a duration of 60 min [32].

Parameters		W0	WT1	WT2	WT3	WT4	WT5
	x ± SD						
**C**	**%**	48.05 ^c^ ± 0.1	48.23 ^c^ ± 0.35	49.12 ^bc^ ± 0.24	52.13 ^b^ ± 0.31	53.94 ^ab^ ± 0.22	55.46 ^a^ ± 0.14
**H**		5.55 ^ab^ ± 0.03	5.87 ^a^ ± 0.03	5.94 ^a^ ± 0.08	4.42 ^c^ ± 0.12	4.06 ^cd^ ± 0.08	3.64 ^d^ ± 0.02
**N**		0.55 ^d^ ± 0.02	1.48 ^a^ ± 0.06	1.26 ^b^ ± 0.05	1.15 ^c^ ± 0.02	1.06 ^c^ ± 0.04	1.30 ^b^ ± 0.06
**Moisture Content**		10.31 ^a^ ± 0.1	9.12 ^b^ ± 0.07	8.74 ^bc^ ± 0.08	8.42 ^bc^ ± 0.1	8.16 ^c^ ± 0.17	7.96 ^c^ ± 0.1
**Ash Content**		3.15 ^b^ ± 0.1	3.17 ^b^ ± 0.1	3.38 ^ab^ ± 0.09	3.52 ^a^ ± 0.1	3.64 ^a^ ± 0.11	3.72 ^a^ ± 0.13
**Volatile Matter**		25.45 ^c^ ± 0.34	23.13 ^d^ ± 0.25	26.92 ^bc^ ± 0.19	31.63 ^b^ ± 0.41	40.27 ^a^ ± 0.21	44.91 ^a^ ± 0.31
**LHV**	**MJ·kg^−1^**	17.51 ^c^ ± 0.25	19.24 ^b^ ± 0.06	20.14 ^b^ ± 0.12	21.39 ^a^ ± 0.22	21.42 ^a^ ± 0.12	21.46 ^a^ ± 0.09
		**S0**	**ST1**	**ST2**	**ST3**	**ST4**	**ST5**
**C**	**%**	45.31 ^d^ ± 0.07	48.61 ^c^ ± 0.15	50.11 ^c^ ± 0.14	52.24 ^b^ ± 0.21	53.44 ^ab^ ± 0.18	55.08 ^a^ ± 0.04
**H**		7.10 ^a^ ± 0.05	5.67 ^b^ ± 0.04	5.06 ^bc^ ± 0.05	4.22 ^c^ ± 0.12	4,09 ^c^ ± 0.08	3.54 ^d^ ± 0.02
**N**		0.15 ^d^ ± 0.01	1.05 ^c^ ± 0.06	1.08 ^c^ ± 0.03	1.18 ^b^ ± 0.02	1.11 ^b^ ± 0.04	1.07 ^a^ ± 0.06
**Moisture Content**		9.18 ^a^ ± 0.12	8.50 ^b^ ± 0.14	7.14 ^bc^ ± 0.14	6.32 ^c^ ± 0.11	5.87 ^c^ ± 0.11	4.52 ^d^ ± 0.1
**Ash Content**		4.56 ^d^ ± 0.12	6.27 ^c^ ± 0.13	6.94 ^c^ ± 0.16	8.66 ^b^ ± 0.1	9.04 ^a^ ± 0.09	9.25 ^a^ ± 0.1
**Volatile Matter**		17.70 ^d^ ± 0.19	20.48 ^c^ ± 0.3	28.74 ^bc^ ± 0.21	36.37 ^b^ ± 0.26	41.23 ^ab^ ± 0.24	47.86 ^a^ ± 0.25
**LHV**	**MJ·kg^−1^**	17.59 ^d^ ± 0.1	18.77 ^c^ ± 0.09	19.04 ^c^ ± 0.17	19.75 ^b^ ± 0.31	20.14 ^b^ ± 0.15	20.96 ^a^ ± 0.31

x—arithmetic mean; SD—standard deviation. Statistically significant differences between marked by different letters in the rows (*p* ≤ 0.05).

**Table 2:** The results of analyses assessing dust from raw and torrefied willow biomass and wheat straw.

Material	P_max_	(*dp/dt*)_max_	LEL—Lower Explosion Limit
	[bar]	[bar·s^−1^]	[g·m^3^]
W0	7.2	261.3	500
WT1	7.0	268.1	500
WT2	7.9	272.4	500
WT3	8.2	279.6	500
WT4	8.6	284.3	250
WT5	9.2	296.6	250
S0	7,3	201,4	500
ST1	7.5	209.6	500
ST2	7.7	214.9	500
ST3	8.1	219.5	500
ST4	8.3	224.3	500
ST5	8.8	231.6	250

**Table 3:** Classes of dust explosivity [42].

Explosivity Class	Value *K_st_*_max_ [bar*s^−1^]
St 1	≤200
St 2	200–300
St 3	>300

## Abbreviations

WT1	willow torrefied at 220 °C
WT2	willow torrefied at 240 °C
WT3	willow torrefied at 260 °C
WT4	willow torrefied at 280 °C
WT5	willow torrefied at 300 °C
S0	raw wheat straw
ST1	straw torrefied at 220 °C
ST2	straw torrefied at 240 °C
ST3	straw torrefied at 260 °C
ST4	straw torrefied at 280 °C
ST5	straw torrefied at 300 °C

## References

[1] Azzuni A., Breyer C. (2020). Global energy security index and its application on national level. Energies.

[2] Nyga-Łukaszewska H., Aruga K., Stala-Szlugaj K. (2020). Energy security of poland and coal supply: Price analysis. Sustainability.

[3] Loeschel A., Moslener U., Ruebellke D.T.G. (2010). Indicators of energy security in industrialised countries. Energy Policy.

[4] (2019). Euracoal Statistics. https://euracoal.eu/info/euracoal-eu-statistics/.

[5] Ymeri P., Gyuricza C., Fogarassy C. (2020). Farmers’ attitudes towards the use of biomass as renewable energy—A case study from southeastern europe. Sustainability.

[6] Lee T., Han E., Moon U.-C., Lee K.Y. (2020). Supplementary control of air–fuel ratio using dynamic matrix control for thermal power plant emission. Energies.

[7] Ratte J., Fardet E., Mateos D., Hery J.S. (2011). Mathematical modelling of a continuous biomass torrefaction reactor: TORSPYD (TM) column. Biomass Bioenergy.

[8] Uslu A., Faaij A.P.C., Bergman P.C.A. (2008). Pre-treatment technologies and their effect on international bioenergy supply chain logistics. Techno-economic evaluation of torrefaction fast pyrolysis and pelletisation. Energy.

[9] Bergman P.C.A., Kiel J.H.A. Torrefaction for biomass upgrading. Proceedings of the 14th European Biomass Conference & Exhibition.

[10] Chen W., Kuo P. (2010). A study on torrefaction of various biomass materials and its impact on lignocellulosic structure simulated by a thermogravimetry. Energy.

[11] Bridgeman T.G., Jones J.M., Shield I., Williams P.T. (2008). Torrefaction of reed canary grass wheat straw and willow to enhance solid fuel qualities and combustion properties. Fuel.

[12] Arias B., Pevida C., Fermoso J., Plaza M.G., Rubeira F., Pis J.J. (2008). Influence of torrefaction on the grindability and reactivity of woody biomass. Fuel Process. Technol..

[13] Medic D., Darr M., Shah A., Potter B., Zimmerman J. (2012). Effects of torrefaction process parameters on biomass feedstock upgrading. Fuel.

[14] Stelte W., Holm J., Sanadi A., Barsberg S., Ahrenfeldt J., Henriksen U. (2011). Fuel pellets from biomass: The importance of the pelletizing pressure and its dependency on the processing conditions. Fuel.

[15] Styks J., Wróbel M., Frączek J., Knapczyk A. (2020). Effect of compaction pressure and moisture content on quality parameters of perennial biomass pellets. Energies.

[16] Popovicheva O., Ivanov A., Vojtisek M. (2020). Functional factors of biomass burning contribution to spring aerosol composition in a megacity: Combined FTIR-PCA analyses. Atmosphere.

[17] Pérez J., Melgar A., Nel Benjumea P. (2012). Effect of operating and design parameters on the gasification/combustion process of waste biomass in fixed bed downdraft reactors: An experimental study. Fuel.

[18] Duan F., Zhang J.-P., Chyang C.-S., Wang Y.-J., Tso J. (2014). Combustion of crushed and pelletized peanut shells in a pilot-scale fluidized-bed combustor with flue gas recirculation. Fuel Process. Technol..

[19] Amyotte P.R. (2014). Some myths and realities about dust explosions. Process Saf. Environ. Prot..

[20] Kauschinger B., Schroeder S. (2016). Uncertainties in heat loss models of rolling bearings of machine tools. Procedia CIRP.

[21] Haghighi Mood S., Hossein Golfeshan A., Tabatabaei M., Salehi Jouzani G., Hassan Najafi G., Gholami M., Ardjmand M. (2013). Lignocellulosic biomass to bioethanol, a comprehensive review with a focus on pretreatment. Renew. Sustain. Energy Rev..

[22] Hawksworth S., Rogers R., Proust C., Beyer M., Zakel S., Gummer J. (2010). Ignition of explosive atmosphere by mechanical equipment. Manchester Hazards XVIII. Process Safety-Sharing Best Practice.

[23] Kakitis A., Nulle I. (2009). Electrostatic biomass mixing. Eng. Rural. Dev..

[24] Tumuluru J.S., Heikkila D.J. (2019). Biomass grinding process optimization using response surface methodology and a hybrid genetic algorithm. Bioengineering.

[25] Dibble C.J., Shatova T.A., Jorgenson J.L., Stickel J.J. (2011). Particle morphology characterization and manipulation in biomass slurries and the effect on rheological properties and enzymatic conversion. Biotechnol. Progr..

[26] Dahn C.J., Dastidar A.G. (2003). Requirements for a minimum ignition energy standard. Process. Saf. Prog..

[27] Randeberg E., Eckhoff R.K. (2006). Initiation of dust explosions by electric spark discharges tr iggered by the explosive dust cloud itself. J. Loss Prev. Process Ind..

[28] Węgrzyn A., Zając G. (2008). Selected aspects of research on energetic effectiveness of plant biomass production technology. Acta Agroph..

[29] Chin K., H’ng P., Go W., Wong W., Lim T., Maminski M., Paridah M., Luqman A. (2013). Optimization of torrefaction conditions for high energy density solid biofuel from oil palm biomass and fast growing species available in Malaysia. Ind. Crops Prod..

[30] Poudel J., Karki S., Oh S.C. (2018). Valorization of waste wood as a solid fuel by torrefaction. Energies.

[31] Werkelin J., Skrifvars B.J., Hupa M. (2005). Ash-forming elements in four Scandinavian wood species. Part. 1. Summer harvest. Biomass Bioenergy.

[32] Bajcar M., Zaguła G., Saletnik B., Tarapatskyy M., Puchalski C. (2018). Relationship between torrefaction parameters and physicochemical properties of torrefied products obtained from selected plant biomass. Energies.

[33] Cashdollar K.L. (2000). Overview of dust explosibility characteristics. J. Loss Prev. Process Ind..

[34] Cordero T., Marquez F., Rodriquez-Mirasol J., Rodriguez J.J. (2001). Predicting heating values of lignocellulosic and carbonaceous materials from proximate analysis. Fuel.

[35] Demirbaş A. (1997). Calculation of higher heating values of biomass fuels. Fuel.

[36] Torrent J.G., Lazaro E.C., Wilén C., Rautalin A. (1998). Biomass dust explosibility at elevated initial pres sures. Fuel.

[37] Holbrow P. (2013). Dust explosion venting of small vessels and flameless venting. Process Saf. Environ. Prot..

[38] Kok M.V., Ozgur E. (2013). Thermal analysis and kinetics of biomass samples. Fuel Process. Technol..

[39] Medina C.h., Phylaktou H.N., Sattar H., Andrews G.E., Gibbs B.M. (2013). The development of an experimental method for the determination of the minimum explosible concentration of biomass powders. Biomass Bioenergy.

[40] Ragland K.W., Aerts D.J., Baker A.J. (1991). Properties of wood for combustion analysis. Bioresour. Technol..

[41] Eckhoff R.K. (2005). Current status and expected future trends in dust explosion research. J. Loss Prev. Process Ind..

[42] Taveau J. (2014). Application of dust explosion protection systems. Procedia Eng..

[43] Arnaldos J., Casal J., Planas-Cuchi E. (2001). Prediction of flammability limits at reduced pressures. Chem. Eng. Sci..

[44] Porowski R., Rudy W., Teodorczyk A. (2012). Analysis of experimental methods for explosion limits of flammable liquids. Fire Saf. Technol..

[45] PN-EN 14034-2—Determination of Explosion Characteristics of Dust Clouds. www.pkn.pl.

